# Butyric Acid Modulates Gut Microbiota to Alleviate Inflammation and Secondary Bone Loss in Ankylosing Spondylitis

**DOI:** 10.3390/biomedicines13010009

**Published:** 2024-12-24

**Authors:** Ke You, Lianjun Yang, Zhihai Su, Jun Shen, Xinyang Fan, Yuanqing Guo, Zhen Yuan, Hai Lu

**Affiliations:** 1 Department of Spine Surgery, The Fifth Affiliated Hospital of Sun Yat-Sen University, Zhuhai 519082, China; yc37634@um.edu.mo (K.Y.); drlianjunyang@163.com (L.Y.); 13265092954@163.com (Z.S.); 13902868105@139.com (Y.G.); 2 Faculty of Health Sciences, University of Macau, Macau 999078, China; 3 Department of Spine Surgery, The Seventh Affiliated Hospital of Sun Yat-Sen University, Shenzhen 518107, China; 13421398163@163.com; 4 Centre of Education Development, South China Normal University, Guangzhou 510006, China; gracexyfan@uic.edu.cn

**Keywords:** ankylosing spondylitis, gut microbiota, short-chain fatty acid, inflammatory response, bone loss

## Abstract

**Background:** Ankylosing spondylitis (AS) is a chronic inflammatory and autoimmune disease that primarily affects the sacroiliac joints and axial skeleton. While the exact pathogenetic mechanism of AS remains unclear, previous reports have highlighted the involvement of genetic factors, immune responses, and gut microbiota dysregulation in the development of this condition. Short-chain fatty acids (SCFAs), which are microbial fermentation products derived from sugar, protein, and dietary fibers, play a role in maintaining the intestinal barrier function and reducing inflammatory responses. The aim of this study was to investigate the therapeutic potential of butyric acid (BA), an important SCFA, in the treatment of AS. **Methods:** To evaluate the anti-inflammatory and anti-bone loss effects of BA, a murine AS model was established using proteoglycan and dimethyl dioctadecyl ammonium (DDA) adjuvants. Various techniques, including an enzyme-linked immunosorbent assay (ELISA), magnetic resonance imaging (MRI), micro-CT, histology, quantitative PCR (qPCR) for intestinal tight junction protein expression, and 16S rDNA sequencing to analyze gut microbiota abundance, were employed to assess the inflammation and bone health in the target tissues. **Results:** The results indicated that BA demonstrated potential in alleviating the inflammatory response in the peripheral joints and the axial spine affected by AS, as evidenced by the reductions in inflammatory infiltration, synovial hyperplasia, and endplate erosion. Furthermore, BA was found to impact the intestinal barrier function positively. Notably, BA was associated with the downregulation of harmful inflammatory factors and the reversal of bone loss, suggesting its protective effects against AS. **Conclusions:** These beneficial effects were attributed to the modulation of gut microbiota, anti-inflammatory properties, and the maintenance of skeletal metabolic homeostasis. This study contributes new evidence supporting the relationship between gut microbiota and bone health.

## 1. Introduction

Ankylosing spondylitis (AS) is a chronic inflammatory and autoimmune disease with a complex etiology, primarily affecting the sacroiliac joints and axial vertebrae. In most patients, back pain is an early symptom due to the localized inflammatory responses in the sacroiliac joint space and lower lumbar region. The aberrant bone fusion of sacroiliac, vertebral, and apophyseal joints often develops later, potentially causing dramatic kyphosis and spine stiffness [1]. The global prevalence of AS was reported as 0.23% in Europe, 0.16% in Asia, 0.31% in North America, 0.10% in Latin America, and 0.07% in Africa [2]. AS pathogenesis involves several risk factors, with human leukocyte antigen (HLA)-B27 reportedly demonstrating a 20.1% heritability in patients with AS [3]. Additionally, the interleukin (IL)-23/IL-17 pathway has particular relevance for AS pathogenesis [4]. Peripheral spondylitis arthritis (pSpA) encompasses a range of joint diseases, including reactive arthritis, psoriatic arthritis, enteritide-associated arthritis, and undifferentiated pSpA. And Parida et al. found that the expression profiles of human leucocyte antigen B27 subtypes and fluid cytokines did not represent a statistical difference between reactive arthritis and undifferentiated pSpA, which also indicated the etiological commonality among the different pSpA subtypes [5]. Notably, recent studies have indicated a correlation between enteritis and peripheral arthritis, with bacterial ileitis observed in AS patients positive for HLA-B27 during ileal biopsies [6]. Similarly, HLA-B27 transgenic rats barely progressed to enteritis and peripheral arthritis under germ-free conditions [7]. Therefore, these latter studies suggest that gut dysbiosis may play important roles during AS pathogenesis. Three processes are involved in AS pathogenesis: inflammation, bone erosion, and new bone formation. A low bone mineral density (BMD) is a prominent feature of AS, with the prevalence of osteoporosis in AS patients ranging from 4% to 58% [8]. Meta analyses have shown an increased risk of vertebral fractures in AS patients, with cervical fractures being more common than thoracic and lumbar vertebrae [9]. Effective treatments, such as tumor necrosis factor (TNF) blockades or non-steroidal anti-inflammatory drugs (NSAIDs), are crucial in controlling the early inflammatory responses. In cases where the pathological changes are uncontrolled, osteotomy (bone shortening) may be indicated for spinal deformity in patients with advanced AS [10]. These therapeutic shortcomings therefore warrant the exploration of novel pathological mechanisms underpinning AS and the identification of new therapies to control the inflammation processes. A recent study reported that anti-TNF–α therapy altered the gut microbiota abundance to alleviate peripheral arthritis in AS mice [11]. However, mechanistic insights on how gut microbiota influence this condition remain unclear.

SCFAs are a class of fatty acids with a carbon atomic number less than 5, and acetate, propionate, and butyrate comprise the highest content in the gut environment. But their contents are easily influenced by complex intestinal conditions, including gut microbiota abundance, gut temperature, pH levels, neuroendocrine factors [12], etc. In the body, SCFAs play a beneficial role in gut homeostasis, such as maintaining the balance of microbiotic abundance, recovering the gut barrier, and reducing the risk of infection [13]. Recently, Zaiss developed the concept of a microbiome–gut–joint axis in rheumatoid arthritis, showing that the interactions between the intestinal mucosal immune and an aberrant local microbiota will affect the synovial joints [14]. During the treatment phase of RA, the continuous oral administration of drugs can influence the abundance of the gut microbiota. *Prevotella* is a Gram-negative bacterium that helps digest protein and carbohydrate, but it can also act as a conditional pathogen to induce various inflammatory diseases. And Scher et al. found that *Prevotella copri* was more abundant in untreated rheumatoid arthritis patients than in healthy individuals [15]. Berberine, a herbal modulator of the gut microbiota with an obvious anti-oxidative stress effect, can alleviate collagen-induced arthritis by raising the abundance of butyrate-producing gut microbiota and reducing the abundance of *Prevotella*, which might function through reducing the product of nitrates and improving the intestinal physiologic hypoxia condition [16]. Meanwhile, Pang et al. found that Freund’s complete adjuvant induced arthritis in rat models appearing as a disturbance of the gut SCFA levels and joint synovial destruction, which can be improved by Atractylodes koreana Kitam, one kind of Chinese herb with beneficial regulation of inflammatory factors and gut microbiota [17]. Bai et al. indicated that consuming resistant starch can produce enough propionate to reduce the levels of Treg cells and raise the expression of IL-10 to improve collagen-induced arthritis [18]. Bone–joint diseases always induce synovial hyperplasia, inflammatory infiltration, and joint structure destruction accompanied with abnormal bone metabolism. Synovium is an important tissue in joint cavities, and its function is to generate enough synovial fluid to lubricate joint movement. Synovial hyperplasia is usually an excessive proliferation of synovial cells and tissues caused by an inflammatory change. In the molecular mechanism, the abnormal activation of synovial fibroblasts in collaboration with mesenchymal cells and immune cells in rheumatoid arthritis are considered to play a central role in joint destruction [19]. Lucas et al. demonstrated that SCFAs and a high-fiber diet improve continuous bone loss in collagen-induced arthritis models, and the additional effect of propionate and butyric acid could alleviate swollen joints and regulate the balance of the osteoblast–osteoclast population. The application of SCFAs has proved the concept of gut–joint axials, but spondylarthritis-related research is still lacking.

Due to the complicated etiology and pathogenesis of AS, researchers have constructed several murine models, including murine progressive ankylosis models, human leukocyte antigen-B27 transgenic models, and proteoglycan-induced spondylitis models [20]. In this study, we utilized a proteoglycan-induced spondylitis mouse model to explore the interaction between the gut microbiota, butyric acid, and AS pathogenesis. By modulating the gut microbiota with butyric acid, we aimed to investigate the relationship between gut dysbiosis and AS. Stool samples were collected for 16S rDNA high-throughput sequencing, and inflammasome component expression was assessed to understand the gut–spondylarthritis relationship. MRI and micro-CT techniques were employed to evaluate the inflammatory changes and bone loss in the study animals

## 2. Materials and Methods

### 2.1. Animal Models

Before the study’s commencement, the study protocol received approval from the ethics committee of the Fifth Affiliated Hospital of Sun Yat-Sen University. All animal studies abided by the National Institutes of Health Guidelines for the Care and Use of Laboratory Animals.

Ninety (90) female retired breeder BALB/c mice aged 6–8 months were obtained from Beijing HFK Bioscience Co., Ltd., Beijing, China. The mice were housed in the Molecular Image Central Animal Laboratory of our institute at 22–25 °C, 60% humidity, and a 12 h light–dark cycle. Two weeks were allocated for the mice to acclimatize to their new environment before the studies commenced. The mice were randomly divided into three groups (n = 10 per group): (1) a control group containing healthy mice, (2) an AS model group which received PBS treatment, and (3) an SCFA group where the AS animals received BA treatment.

The construction of the AS mouse model was previously described, namely a triple intraperitoneal injection of proteoglycan and DDA into female BALB/c mice. Briefly, 100 µg of proteoglycan lyophilized powder (Sigma, St. Louis, MO, USA) and 20 mg of DDA adjuvant (Sigma) were mixed in 200 µL of normal saline to generate an emulsion. Then, the mice received 200 µL of intraperitoneal injections of this emulsion at 0, 3, and 6 weeks. The SCFA group received the gavage treatment at 10 weeks via PBS solution supplemented with BA (150 mM, Sigma) for 2 weeks as previously described [21], when the AS group were gavaged with PBS only. The experimental schedule lasted 14 weeks as in our previous work [22].

### 2.2. Arthritis Score Evaluation and Histology

The arthritis severity was previously described using a classical scoring system; grade 0 = normal; 1 = slight redness or swelling in a paw; 2 = moderate redness or swelling involving more than one paw; 3 = obvious erythema or swelling in the paws and ankles; 4 = severe swelling of an entire paw and movement disorder [23]. From the third intraperitoneal injection to tissue harvesting, two independent researchers measured the four-paw arthritis scores weekly and accumulated the total scores for each mouse (the maximum score was 16).

Upon euthanasia, the hind paws, spine, intestine, and colon were harvested and fixed in 4% phosphate-buffered paraformaldehyde for 1 week at room temperature. The tissue attached to the bone samples was decalcified in 10% Titriplex ethylene diamine tetraacetic acid (EDTA) for 3–4 weeks. Then, the samples were dehydrated in a graded ethanol series, embedded in paraffin, serially sectioned to generate 5 μm slices, and stained with hematoxylin-eosin (HE). Images were acquired and edited using a pathological section scanner (3DHISTECH, Budapest, Hungary).

### 2.3. Enzyme-Linked Immuno Sorbent Assay (ELISA)

After 2 weeks of BA administration, the mice were anesthetized with isoflurane, whole blood was collected from the eyeballs, and the serum was separated via centrifugation at 2000× *g* for 10 min. We collected 100 µL serum samples from the control group, AS group, and BA group (5 serum samples per group). All the serum samples were diluted 5 times according to the manufacturer’s instructions for each specific ELISA kit (ABclonal Technology TM, Wuhan, China). The enzyme-labeled plate was pre-coated with anti-mouse TNF-α, IL-17, IL-23, IL-1β, IL-18, IL-10, ALP, and RANKL antibody, which were the primary antibodies. By adding 5 times the diluted samples and standards into the enzyme-labeled plate, the above cytokines will bind to the specific monoclonal antibody. The plates were covered with sealing plate film and incubated at 37 °C for 30 min. After washing away the free sample components, enzyme-labeled antibody was added, which were the secondary antibody and cover with sealing plate film followed by incubation at 37 °C for 30 min. The unbound enzyme-labeled antibodies were washed away, and chromogenic agent A(H_2_O_2_), chromogenic agent B(TMB), and termination solution were added successively. Finally, the OD values were measured at 450 nm following the previous experiment method [22].

### 2.4. Quantitative Real-Time Polymerase Chain Reaction (qRT-PCR)

RNA extraction and quantitative real-time PCR (qRT-PCR) analysis were performed using the Trizol reagent, and the total RNA was extracted from the colon samples. Firstly, the First Strand cDNA Synthesis Kit (Summer Bio, Beijing, China, Cat SUM73061) was used to convert 1 μg of the total RNA into cDNA. Finally, these samples were run using a standard SYBR Green qPCR protocol as previously described [24]. The amplification condition was controlled as three procedures following denaturation: 95 °C for 30 s and annealing; 50 °C for 30 s and extension; 70 °C for 30 s. The primer sequences of the target genes included two types of intestinal tight junction proteins: zonula occludens-1 (ZO-1) and Occludin (Table 1). The relative expression of the above genes was normalized using β-actin.

### 2.5. DNA Extraction from Stool Samples and 16S Ribosomal Deoxyribonucleic Acid (rDNA) High-Throughput Sequencing

DNA extraction from stool samples was performed using the E.Z.N.A.^®^ Stool DNA Kit (D4015, Omega, Inc., Norcross, GA, USA) according to the manufacturer’s instructions. The reagent which was designed to uncover DNA from trace amounts of sample has been shown to be effective for the preparation of the DNA of most bacteria. Nuclease-free water was used as a blank. The total DNA was eluted in 50 µL of elution buffer and stored at −80 °C until PCR (LC-Bio Technology Co., Ltd., Hang Zhou, Zhejiang Province, China).

The V3-V4 region of the 16S rRNA gene provides a balance between taxonomic resolution and diversity coverage in bacterial and archaeal communities, which contain conserved regions for primer binding and variable regions offering species-level resolution. An optimal read length for sequencing accuracy and coverage in the V3-V4 region is well suited for high-throughput sequencing platforms like Illumina [25]. The V3-V4 region of the prokaryotic (bacterial and archaeal) small-subunit (16S) rDNA gene was amplified using 341F (5′-CCTACGGGNGGCWGCAG-3′) and 805R (5′-GACTACHVGGGTATCTAATCC-3′) primers to characterize the microbial composition of stool samples with a balance of taxonomic resolution and sequencing efficiency [26]. Primer 5′ ends were tagged with specific barcodes for the samples and universal sequencing primers. PCR amplification was performed in a 25 µL total volume containing 25 ng of template DNA, 12.5 µL of PCR Premix, 2.5 µL of each primer, and PCR-grade water to 25 µL. The PCR conditions consisted of an initial denaturation at 98 °C for 30 s, followed by 32 cycles of denaturation at 98 °C for 10 s, annealing at 54 °C for 30 s, extension at 72 °C for 45 s, and a final extension at 72 °C for 10 min. The PCR products were confirmed using 2% agarose gel electrophoresis. Throughout the DNA extraction process, ultrapure water instead of a sample solution was used as a negative control to exclude false-positive PCR results. The PCR products were purified using AMPure XT beads (Beckman Coulter Genomics, Danvers, MA, USA) and quantified via a Qubit assay (Invitrogen, Carlsbad, CA, USA). Amplicon pools were prepared for sequencing, with the amplicon library size and quantity assessed on an Agilent 2100 Bioanalyzer (Agilent, Santa Clara, CA, USA) and Library Quantification Kit for Illumina (Kapa Biosciences, Wilmington, MA, USA), respectively. Libraries were sequenced using the NovaSeq PE250 platform.

The samples were sequenced on an Illumina NovaSeq platform according to the manufacturer’s recommendations (LC-Bio). Paired-end reads were assigned to the samples based on unique barcodes. Reads were truncated by severing the barcode and primer sequence. Adobe Flash Player (FLASH) assists in improving the read length and sequence coverage, enhancing the accuracy of downstream analysis. FLASH was utilized for merging the paired-end reads generated by the Illumina sequencing platform. It aligns overlapping paired-end reads to reconstruct full-length sequences for downstream analysis. Fqtrim helps to improve the accuracy of the downstream analysis by removing sequencing artifacts, adapter sequences, and low-quality reads, ensuring that only reliable data are used for further processing. Fqtrim (v0.94) was used for the quality filtering of the raw reads to obtain high-quality clean tags. Specific filtering conditions were applied to retain the reads that met the quality thresholds. DADA2 offers high-resolution sequence analysis, enabling the detection of subtle variations in microbial communities. DADA2 was employed for sequence dereplication to obtain a feature table and feature sequences after dereplication. QIIME2 is a comprehensive bioinformatics platform for microbiome analysis, offering a suite of tools for accurate diversity assessment, taxonomic classification, and functional prediction, facilitating robust and reproducible microbiome research. QIIME2 was used for calculating the alpha and beta diversity indices, taxonomy assignment, and generating visualizations. SILVA and NT-16S databases are widely recognized references for prokaryotic ribosomal RNA sequences, enabling accurate taxonomic assignment and phylogenetic analysis in microbiome studies. The SILVA and NT-16S databases were used for sequence alignments and species annotations, providing reference databases for the taxonomic classification and phylogenetic analysis.

### 2.6. Magnetic Resonance Imaging (MRI)

Following anesthetization with isoflurane via a nose cone, vital signals including heart rate and respiratory rate were synchronously detected. The animal was placed inside the MRI scanner, ensuring that the arthritic and spinal area was within the imaging range. MRI sequences were modulated as follows: T2_TurboRARE_Coronal_fws [echo time (TE) 32 ms; repetition time (TR) 1600 ms; and slice thickness 0.5 mm] and T2_TurboRARE_Axial_fws [echo time (TE) 36 ms; repetition time (TR) 1600 ms; and slice thickness 0.5 mm]. After identifying the region of interest (ROI) from the peripheral joints and axial spine, image analysis software (RadiAnt DICOM Viewer, v4.2.1) was used to evaluate the inflammatory swell signal among the three groups as previously described [27].

### 2.7. Micro-Computed Tomography (Micro CT)

After 14 weeks, femur samples from the different groups were harvested to be fixed in 4% paraformaldehyde solution and analyzed via micro-CT as previously described [28]. The detected parameter was set to 50 kV and 160 µA at a 12.0 µm resolution. Three types of imaging software (NRecon, CTAn v1.9, and CTVol v2.0) were used to analyze the distal femoral metaphyseal trabecular bone. NRecon was used for reconstructing and visualizing the 3D micro-CT images. CTAn v1.9 was employed for the quantitative analysis of the trabecular bone parameters. CTVol v2.0 was utilized for volumetric visualization and analysis of the trabecular bone structure. We selected some ROI to establish two-dimensional morphometric and three-dimensional histomorphometry analysis. Finally, these cross-sectional images of the distal femora were employed to calculate the bone loss relative index including BV/TV, Tb. N, Tb. Sp, and Connectivity.

### 2.8. Statistical Analysis

Studies were independently performed and repeated three times or more. SPSS 22.0 software (SPSS Inc., Chicago, IL, USA) was used to analyze all the data; it is a widely used statistical software package known for its user-friendly interface and robust analytical capabilities. One-way analysis of variance (ANOVA) was used to compare the differences between the groups; it is a statistical test used to compare the means across multiple groups to determine if there are statistically significant differences among them. The Kruskal–Wallis rank sum test was used to calculate the microbial proportions. *p* < 0.05 was considered statistically significant.

## 3. Results

### 3.1. SCFA Ameliorates the Pathological Change of Gut Tissue and Improves the Intestinal Mucosal Barrier Function in AS Mice

**Figure 1:**
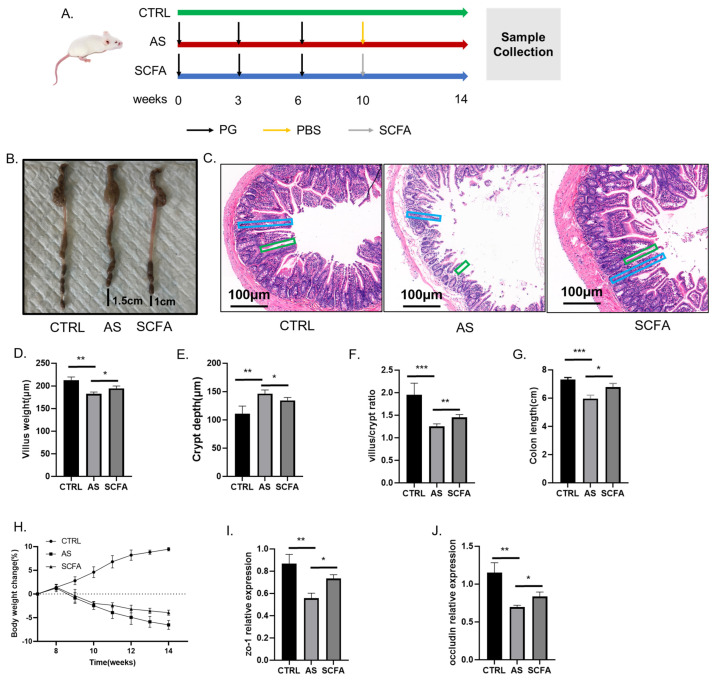
SCFA ameliorates the pathological change of gut tissue and improves intestinal mucosal barrier function in AS mice. Study design (**A**). Colon tissues in groups (**B**). Ileum tissues were stained with H&E, blue box represents crypt depth and green box represents villus height. Scale bar is 100 μm (**C**), statistical data of villus height (**D**), crypt depth (**E**), and villus/crypt ratio (**F**). Colon length (**G**). Body weight change in groups (**H**). mRNA relative expression of ZO-1 (**I**) and Occludin (**J**). Data are expressed as mean ±SD from ten mice/group. *** indicates *p* < 0.001, ** indicates *p* < 0.01, and * indicates *p* < 0.05. Abbreviations: PG—proteoglycan; PBS—phosphate buffer solution; SCFA—short-chain fatty acid; CTRL—control; AS—ankylosing spondylitis.

We constructed a murine AS model using a triple schedule intraperitoneal injection strategy (Figure 1A) and established SCFA intervention conditions in the AS mice using a 2-week BA treatment schedule. Colon tissue from the AS group was 1.5 cm shorter on average than that of the control group (Figure 1B), while the SCFA group could change the pathological condition, and the quantitative statistical results for colon length are shown in Figure 1G. From H&E staining, which represents the gut mucosal change during modeling, we found that normal dense and intact villi appeared in the control group, but in the AS group, there appeared an increased crypt depth and decreased villus height, and the supplement of SCFAs can alleviate the disrupted gut structure and increase the villus/crypt ratio (Figure 1D–F). Influenced by the gut nutrient condition, the weight levels demonstrated a similar change (Figure 1H). In addition, the qRT-PCR results indicated that the expression of tight junction proteins (ZO-1, Occludin) in the AS mice was downregulated obviously compared with the control group and SCFA group, which can reflect the gut barrier dysfunction indirectly (Figure 1I,J).

### 3.2. SCFA Attenuates Peripheral Arthritis and Axial Spondylitis Severity in AS Mice

**Figure 2:**
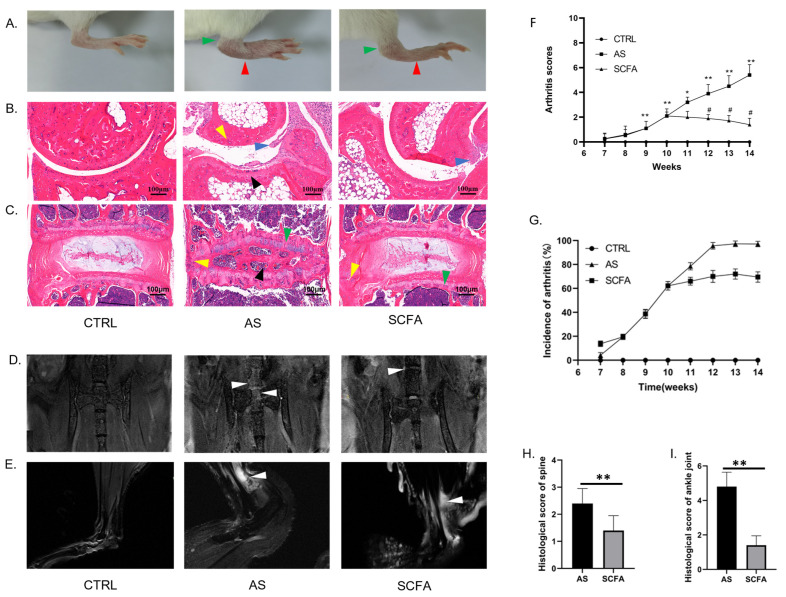
SCFA attenuates peripheral arthritis and axial spondylitis severity in AS mice. Representative hind paw in groups (**A**). Histological ankle changes, green arrow represents ankle swelling and red arrow represents paw red swelling (**B**) (hematoxylin and eosin (H&E) staining). The AS group was characterized by inflammatory infiltration (black arrow), synovial hyperplasia (blue arrow), and pannus formation (yellow arrow). Histological spine changes (**C**) (H&E staining). AS animals were characterized by inflammatory infiltration at the disc periphery (yellow arrow), abnormal bone matrix formation (black arrow), and cartilage endplate damage (green arrow). Magnetic resonance imaging identified characteristic inflammatory signal (white arrows) in spine (**D**) and ankle joints (**E**). Arthritis scores (**F**). Arthritis incidence (**G**). Histological score of spine (**H**) and ankle joints (**I**) (** *p* < 0.01, * *p* < 0.05 AS group vs. SCFA group alone, ^#^ *p* < 0.05, SCFA group vs. Control group alone). Abbreviations: SCFA—short-chain fatty acid; CTRL—control; AS—ankylosing spondylitis.

To evaluate the severity of AS pathology, the swelling symptoms and arthritis scores were recorded in the study groups. The control group did not develop arthritis, whereas the AS animals displayed red swelling at the ankle joints or paws after three times of abdominal immunity. The SCFA groups displayed similar pathological features as the AS animals before treatment, and the SCFA administration could ease the inflammatory change (Figure 2A). Meanwhile, the arthritis scores (Figure 2F) and the incidence of arthritis (Figure 2G) were analyzed quantitatively among the three groups. We used H&E staining to evaluate the histological features of the ankles and vertebrae across the groups. There were no obvious inflammatory signs, e.g., synovial hyperplasia and inflammatory infiltration were observed in the control group. However, pathological changes were recorded in the AS group, comprising synovial hyperplasia, inflammatory infiltration, pannus formation at the ankle (Figure 2B), inflammatory infiltration, cartilage endplate damage, and abnormal bone matrix formation in the intervertebral discs (Figure 2C). When compared with this group, the SCFA administration appeared to alleviate the inflammation reactions and bone destruction in the SCFA group. As a chronic disease, early AS progression is difficult to detect; however, sacroiliac arthritis is an important diagnostic criterion. We used MRI to determine the inflammatory signals across the groups (Figure 2D,E). The control group did not display any related inflammatory signals via MRI. The AS group exhibited vertebral adjacent tissues and joint tissues within the inflammation, suggesting that the early detection of peripheral arthritis and axial spondylitis is beneficial for evaluating AS pathological changes.

### 3.3. SCFA Increases Anti-Inflammatory Cytokine Expression, but Reduces the Expressions of Pro-Inflammatory Cytokines

**Figure 3:**
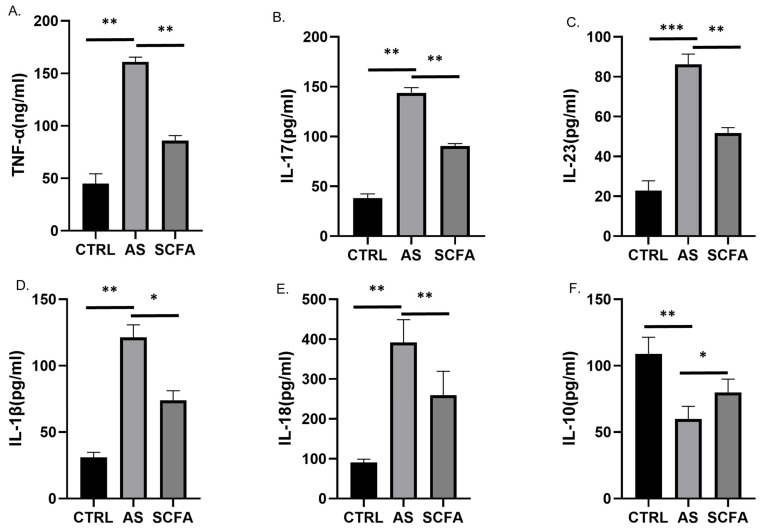
SCFA increases anti-inflammatory cytokine expression, but reduces the expression of pro-inflammatory cytokines. Plasma pro-inflammatory and anti-inflammatory cytokine levels, including TNF-α, IL-17, IL-23, IL-1β, IL-18, and IL-10 were determined via enzyme-linked immunosorbent assay (ELISA) (**A**–**F**). Values are presented as the means ± standard deviation from ten mice/group. *** indicates *p* < 0.001, ** indicates *p* < 0.01, and * indicates *p* < 0.05. Abbreviations: SCFA—short-chain fatty acid; CTRL—control; AS—ankylosing spondylitis; TNF-α—tumor necrosis factor-α; IL—interleukin.

As demonstrated by the ELISA data, when compared to the control group, multiple pro-inflammatory cytokines (IL-17, IL-23, IL-1β, IL-18, and TNF-α) were increased in the AS groups, and IL-10, one kind of anti-inflammatory cytokine, was decreased. During the 2-week SCFA treatment, the above inflammatory levels were modulated (Figure 3A–F).

### 3.4. SCFA Reverses the Condition of Bone Loss and Increases Bone Mineral Density in AS Mice

**Figure 4:**
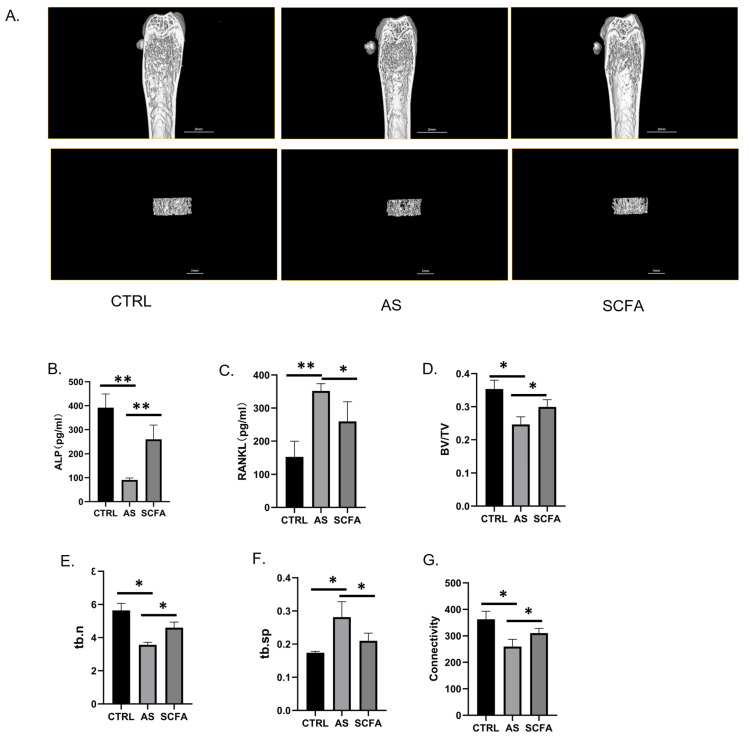
SCFA reverses the condition of bone loss and increases bone mineral density in AS mice. Representative pictures of micro-CT scans from the three groups (**A**). Plasma bone metabolism cytokine levels, including ALP (**B**) and RANKL (**C**) were determined via enzyme-linked immunosorbent assay (ELISA). Bone loss relative index including BV/TV (**D**), Tb. N (**E**), Tb. Sp (**F**), and Connectivity (**G**). Values are represented as the mean ± standard deviation (SD), ** indicates *p* < 0.01, and * indicates *p* < 0.05. Abbreviations: SCFA—short-chain fatty acid; CTRL—control; AS—ankylosing spondylitis; ALP—alkaline phosphatase; RANKL—receptor activator of nuclear factor-κ B ligand; BV/TV—bone volume over total volume; Tb.n—trabecular numbers, Tb.sp—trabecular separation.

Micro-CT scanning was used to evaluate the mouse distal femur bone loss in the three groups, and some coronal and axial dissected sections are displayed (Figure 4A). Then, we selected the ROI to analyze the bone mineral density and bone microarchitecture quantitatively, which can represent the bone remodeling affected by AS modeling. It was demonstrated that these bone remodeling indexes (BV/TV, Tb. N, and Connectivity) (Figure 4D,E,G) decreased in the AS mice, and the bone loss index (Tb. Sp) (Figure 4F) increased compared with the other groups. Osteoclastic factors are responsible for bone resorption or loss, while osteoblastic factors contribute to the regulation of osteoblast activity and bone formation. Monitoring two kinds of factors provides insights into the dynamic process of bone remodeling [29]. Interestingly, SCFAs can reverse bone loss and increase the mineral density in trabecular bone. Meanwhile, two types of bone metabolism serum factors were detected by using the ELISA kit (ALP, RANKL). The AS group demonstrated the specific expression of high osteoclastic factors and low osteoblastic factors, while the other two groups exhibited a contrary trend (Figure 4B,C).

### 3.5. SCFA Administration Modulated the Diversity and Composition of Gut Microbiota in AS Mice

**Figure 5:**
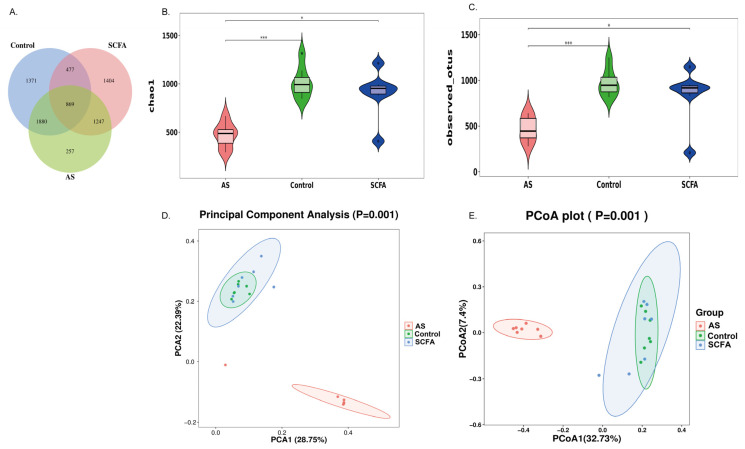
SCFA administration modulated diversity and composition of gut microbiota in AS mice. Venn diagram showing operational taxonomic units (OTUs) in groups (**A**). Alpha diversity showing the Chao1 index (**B**), Observed species (**C**), *** indicates *p* < 0.001, and * indicates *p* < 0.05. Beta diversity at the phylum level in three groups was assessed via PCA (Principal Component Analysis) and principal coordinate analysis (PCoA) of weighted UniFrac (**D**,**E**). Abbreviations: SCFA—short-chain fatty acid; CTRL—control; AS—ankylosing spondylitis.

To distinguish the microbial genes between the groups, operational taxonomic unit (OTU) data displayed via a Venn diagram demonstrated that 869 of 7505 OTUs were common to all three groups, with 1371 distinct microbial genes in the control group, 1404 in the SCFA group, and 257 in the AS group (Figure 5A). The Chao1 index is a non-parametric estimator used to estimate the species richness within a microbial community based on the number of operational taxonomic units (OTUs) present, and a higher Chao1 value suggests greater microbial diversity and richness in the community. The Observed index represents the actual number of distinct microbial species (OTUs) observed in a given sample without considering rare species [30]. The α-diversity of the gut microbiota among the three groups was evaluated by employing the Chao1 and Observed indexes, and the results indicated that the SCFA treatment modulated the microbiota abundance. Briefly, compared with the AS group, the Chao 1 (Figure 5B) and Observed (Figure 5C) indexes showed an obvious increasing trend.

The β-diversity quantifies the differences in species composition between the microbial communities in the different samples or groups, which can be measured using statistical metrics such as PCA and PCoA. PCA (Principal Component Analysis) is a multivariate statistical technique used to identify the patterns and relationships in high-dimensional data by reducing its dimensionality while preserving the variation, and PCoA (principal coordinate analysis of weighted UniFrac) is a method for visualizing and interpreting the similarities or dissimilarities between microbial communities based on phylogenetic information [31]. PCA (Figure 5D) and PCoA (Figure 5E) were used to evaluate the similarities of the gut microbiota among the three groups, and the result showed a significant difference regarding the AS group compared with the other group, but the species composition of the SCFA group was similar to that of the control group.

**Figure 6:**
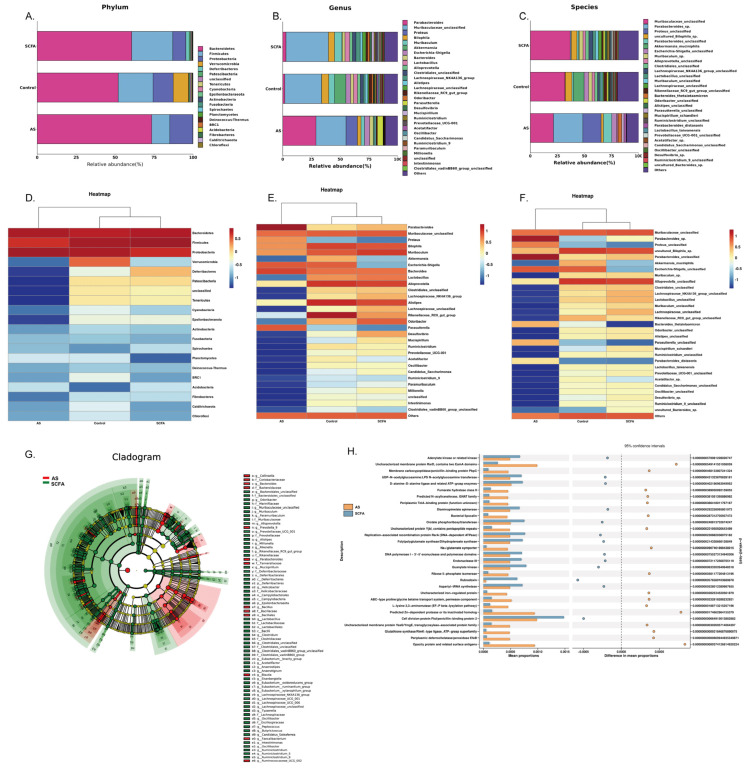
Comparison of the gut microbiota taxa and functional prediction in the three groups. Relative abundance analyses of gut microbiota composition at the Phylum (**A**), Genus (**B**), and Species (**C**) levels in three groups. Heatmap showing the differences between three groups at the Phylum (**D**), Genus (**E**), and Species (**F**) levels. LEfSe analysis of the gut microbiota between AS and SCFA groups (**G**). Functional predictions were performed based on the Kyoto Encyclopedia of Genes and Genomes (KEGG) functional categories between AS and SCFA groups (**H**). Abbreviations: SCFA—short-chain fatty acid; CTRL—control; AS—ankylosing spondylitis.

Feature taxonomy was performed to evaluate the composition of the gut microbiota among the three groups. The phylum abundance (Figure 6A) indicated that the species of gut microbiota were dominated by *Bacteroidetes* and *Firmicutes* in the control group and SCFA group, but the AS group showed an increased abundance of *Bacteroidetes* (73.44%) and decreased *Firmicutes* (11.58%) compared with the abundance of *Bacteroidetes* (51.99%) and *Firmicutes* (22.33%) in the control group and the abundance of *Bacteroidetes* (60.59%) and *Firmicutes* (26.38%) in the SCFA group. Meanwhile, the abundance of *Proteobacteria* (14.78%) was increased compared with the control group (12.94%) and SCFA group (8.38%). On the genus (Figure 6B) and species (Figure 6C) levels, the gut microbiota abundance was dominated by *Parabacteroides* in the AS group, but there was mostly *Muribaculaceae* in the control and SCFA groups.

Heatmaps are graphical representations used to visually represent the relative abundance or expression levels of different taxa or features within microbial communities. We also present some heatmaps to show the gut microbiota difference at the phylum (Figure 6D), genus (Figure 6E), and species (Figure 6F) levels. Clustering analysis showed that the relative abundance of *Parabacteroides* and *Proteus* was raised obviously compared with the control group, while the abundance of *Proteus* and *Muribaculum* decreased compared with the control group, but administration of SCFAs could modulate the abundance of the above gut microbiota.

In order to compare the significant difference between the AS and SCFA groups, LEfSe analysis (Figure 6G) was performed to explore the key communities of gut microbiota. Briefly, samples from the AS and SCFA groups were categorized into distinct classes to compare the differential abundance of microbial taxa between the groups. LEfSe employs non-parametric tests to identify microbial taxa that show significant differences in abundance between the AS and SCFA groups. After identifying the statistically significant features, LEfSe utilizes LDA to assess the effect size of these features, ranking them based on their discriminative power. LEfSe generates a cladogram that is used to illustrate the hierarchical differences in the abundance of microbial taxa between the AS and SCFA groups. The results demonstrated that *Collinsella*, *Bacteroides*, *Prevotella*, *Parabacteroides*, and *Bacillaceae* were enriched in the AS group, and *Muribaculaceae* were obviously higher in number in the SCFA group. The PICRUSt program (Figure 6H) was used to analyze the functional pathway, and the STAMP variation analysis indicated the high expression of adenylate kinase and cell division protein. Ftsl appeared in the gut microbiota of the AS group, while in the SCFA group, the genes were related to the membrane protein Rard, Opacity protein, and Zn-dependant protease. LEfSe analysis is applied when there is a need to pinpoint the key microbial communities associated with specific conditions or treatments. The PICRUSt program is utilized when investigating the potential functional pathways and metabolic capabilities of microbial communities. STAMP was chosen to analyze the variations in gene functions or pathways derived from metagenomic data [32].

## 4. Discussion

**Figure 7:**
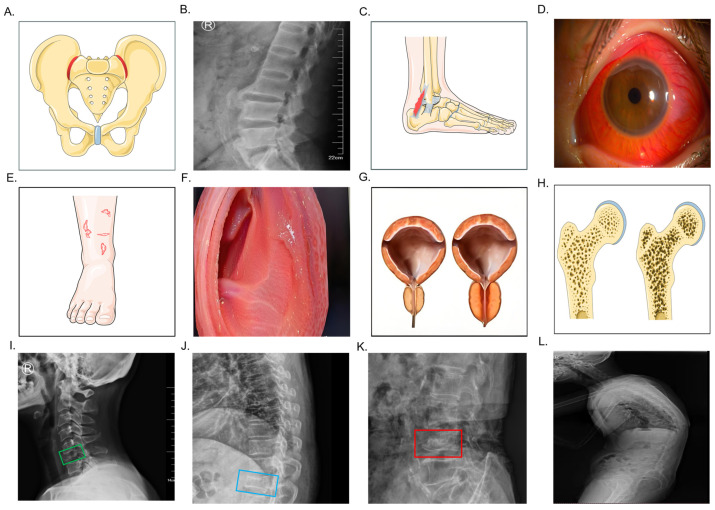
Clinical symptoms and complications of AS: sacroiliitis (**A**); spinal syndesmophytes (**B**); enthesitis (**C**); anterior uveitis (**D**); psoriasis (**E**); enteropathy (**F**); prostatitis (**G**); osteoporosis (**H**); cervical vertebral fracture, green box represents fracture region (**I**); thoracic vertebral fracture, blue box represents fracture region (**J**); lumbar vertebral fracture, red box represents fracture region (**K**); and kyphosis (**L**).

Inflammatory tissue damage is a pervasive feature in the lives of individuals with ankylosing spondylitis (AS), a complex chronic inflammatory disease. The clinical manifestations of AS encompass both articular and extra-articular symptoms. While most AS patients experience articular disease characterized by sacroiliitis, spinal syndesmophytes, dactylitis, and enthesitis, there are also reported cases of extra-articular symptoms such as enteropathy, anterior uveitis, osteoporosis, and pathological fractures (Figure 7) [33]. In recent years, there has been a burgeoning interest in understanding the role of the gut microbiota in the pathogenesis of AS [34]. Emerging evidence suggests that pro-inflammatory cytokines like IL-17/TH17 and gut microbiota are pivotal players in the progression of AS [35]. This study aimed to investigate the potential therapeutic effects of butyrate acid, a short-chain fatty acid, in modulating the gut microbiota to alleviate inflammation and secondary bone loss in AS.

Butyric acid, a short-chain fatty acid produced via gut microbiota fermentation of dietary fiber, has gained attention for its potential role in modulating the immune responses and inflammation. Studies have demonstrated that butyric acid can regulate the differentiation and function of immune cells, suppress pro-inflammatory cytokine production, and promote regulatory T cell generation [36]. These immunomodulatory effects suggest that butyric acid may have therapeutic benefits in autoimmune and inflammatory conditions, including AS. Recent preclinical studies have investigated the impact of butyric acid supplementation on experimental models of AS, showing potential anti-inflammatory and disease-modifying effects [37]. Clinical trials evaluating the efficacy and safety of butyric acid in AS patients are underway, aiming to establish its therapeutic potential in a clinical setting. The preliminary findings suggest that butyric acid supplementation may improve disease activity, reduce inflammation, and enhance the clinical outcomes in AS patients [38]. Further research is needed to elucidate the optimal dosing, administration, and long-term effects of butyric acid treatment in AS.

*Bacteroidetes* and *Firmicutes* are the key players in maintaining gut homeostasis and influencing the host immune responses. In healthy individuals, a balanced ratio of Bacteroidetes to *Firmicutes* is associated with gut health and immune tolerance [39]. However, dysregulation of this balance, characterized by decreased *Firmicutes* and increased *Bacteroidetes* abundance, has been observed in various inflammatory conditions, including AS. The dominance of *Bacteroidetes* and *Firmicutes* in the gut microbiota of AS patients compared to healthy controls may reflect a dysbiotic state associated with altered immune activation and inflammatory responses [15]. The interplay between SCFAs and gut microbiota composition, particularly the balance of *Bacteroidetes* and *Firmicutes*, is crucial for immune homeostasis and overall health. Butyric acid may increase *Bacteroidetes* and decrease *Firmicutes* and could involve its regulatory effect on the gut microbiota. By modulating the structure and function of the gut microbiota, butyric acid may create an environment more conducive to the preferential increase in Bacteroidetes, while reducing the presence of Firmicutes [40].

The main focus of this study was to investigate the potential therapeutic advantages of butyric acid, a short-chain fatty acid, in regulating the gut microbiota to reduce inflammation and mitigate secondary bone loss in individuals with ankylosing spondylitis (AS). Through the specific targeting of the gut microbiota using butyric acid, our aim was to assess its effectiveness in alleviating inflammation and maintaining bone health in AS patients. This study underscores the promising potential of novel therapeutic interventions that leverage the interplay between the gut microbiota, inflammation, and bone health within the context of AS. Based on our ELISA data (Figure 3), we observed significant increases in multiple pro-inflammatory factors (IL-17, IL-23, TNF-α, IL-1β, and IL-18) in the serum of mice with ankylosing spondylitis (AS) compared to the control group (*p* < 0.05). The administration of short-chain fatty acids (SCFAs) was associated with a reduction in the overexpression of these inflammatory factors (Figure 3A–E) and an increase in the expression of anti-inflammatory cytokines (Figure 3F). In AS, there is typically a suppression of tight junction proteins, indicating dysfunction in the gut barrier. Through our observations, SCFAs were able to modulate bone metabolism cytokines as well. Specifically, from some research of arthritis, butyric acid (BA) was found to downregulate the osteoclastic cytokine receptor activator of nuclear factor kappa-B ligand (RANKL) and promote the secretion of alkaline phosphatase (ALP), an osteoblastic cytokine [41]. This dual action of downregulating bone-resorbing factors (Figure 4C) and promoting bone-forming factors (Figure 4B) suggests a beneficial effect of BA on bone health in the context of AS. Overall, these findings suggest that SCFAs, particularly BA, may play a role in mitigating inflammation, maintaining gut barrier function, and regulating bone metabolism in AS.

Detecting and understanding the pathological processes in ankylosing spondylitis (AS) pose significant challenges, as the inflammation leading to bone erosion can persist over many years. This chronic nature makes animal models essential for experimental research in this field. Given the importance of genetic factors, inflammation, and autoimmunity in AS, various animal models have been developed, including HLA-B27 transgenic models, inflammation-associated models, and models associated with ankylosing enthesitis [20]. In a previous study utilizing HLA-B27 transgenic rats, researchers sought to explore the potential link between AS and gut dysbiosis, focusing on evaluating the inflammatory changes in ileal and joint tissues but not the spine [42]. In our current study, we opted to induce an inflammation-associated AS model using proteoglycan, mimicking the pathological progression observed in AS patients. This model initiates with peripheral joint symptoms such as synovitis, synovial hyperplasia, and cartilage erosion, ultimately leading to the destruction of axial vertebrae in later stages. Histological examination with hematoxylin and eosin staining revealed significant changes in the ankle and intervertebral disks (Figure 2B,C), while MRI scans exhibited early signs of sacroiliitis and spondylitis in our model (Figure 2D,E). Additionally, micro-CT imaging demonstrated secondary bone loss. These findings collectively demonstrate the relevance of our AS model in replicating the key pathological features observed in AS patients, providing valuable insights for further research in this area.

The anti-inflammatory properties of butyrate have been extensively documented across a range of inflammatory conditions, including autoimmune diseases [43], inflammatory bowel disease [44], metabolic disorders [45], neurodegenerative diseases [46], and colorectal cancer [47], and supplementation with *Clostridium Butyricum* has shown potential protective effects in various studies. In our research, the administration of butyrate led to a significant decrease in the circulating levels of inflammatory cytokines, highlighting its robust anti-inflammatory effects. Moreover, our analysis of gut function revealed that butyrate treatment was associated with improvements in parameters such as body weight (Figure 1H), colon length (Figure 1B,G), and intestinal barrier integrity, including crypt depth (Figure 1E) and villus height (Figure 1D), all of which suggest positive effects on gut health mediated by short-chain fatty acids (SCFAs). Tight junction proteins play a crucial role in regulating the intestinal barrier function, reducing intestinal permeability, and preventing gut leakage and bacterial translocation [48]. Previous findings have indicated that psychological stress can lead to the suppression of tight junction protein expression, exacerbating gut dysbiosis [49]. In our study, we investigated the expression of the tight junction proteins ZO-1 (Figure 1I) and Occludin (Figure 1J) using quantitative real-time polymerase chain reaction (qRT-PCR). The results demonstrated a significant downregulation of both proteins in the AS groups, indirectly indicating impaired gut barrier function during the progression of AS. Therefore, supplementation with SCFAs appeared to ameliorate AS-induced gut barrier damage by modulating the tight junction protein expression, offering a potential avenue for therapeutic intervention to restore the gut barrier integrity in AS.

One of the significant complications associated with ankylosing spondylitis (AS) is secondary bone loss, a condition that substantially contributes to functional limitations and disability [50]. Our research findings provide insights into the beneficial effects of butyrate supplementation in mitigating bone loss in AS through various mechanisms. Firstly, butyrate was observed to enhance the expression and activity of osteoblastic cytokines (Figure 4B), particularly alkaline phosphatase (ALP), which plays a crucial role in bone formation. Additionally, butyrate demonstrated the ability to inhibit the overexpression of osteoclastic cytokines (Figure 4C) such as the receptor activator of nuclear factor kappa-B ligand (RANKL), which is involved in bone resorption. Secondly, butyrate supplementation was found to alleviate the levels of several pro-inflammatory cytokines, including IL-1β (Figure 3D) and IL-18 (Figure 3E), which have been associated with inflammatory bone loss in previous studies [51]. By modulating the factors involved in bone metabolism and suppressing inflammatory cytokines, butyrate effectively prevents excessive bone erosion and the subsequent bone loss in individuals with AS. These dual actions on bone formation and resorption pathways highlight the potential of butyrate as a therapeutic agent for preserving bone health and countering the detrimental effects of inflammation on bone tissue in AS.

The role of the gut microbiota in regulating systemic immune responses and overall health is paramount, with gut dysbiosis—characterized by an imbalance in the gut microbial composition—being implicated in the pathogenesis of autoimmune diseases, including ankylosing spondylitis (AS) [52]. Consequently, targeting the gut microbiota through butyrate supplementation is emerging as a novel and promising therapeutic approach for managing AS and its associated complications. Several studies have underscored a strong connection between the gut microbiota composition and osteoporosis, with the ratio of *Bacteroidetes* to *Firmicutes* playing a crucial role in maintaining gut microbial balance and health [53]. Our research has revealed an imbalance in the *Bacteroidetes* and *Firmicutes* ratio in AS, and supplementation with short-chain fatty acids (SCFAs) was effective in restoring this abnormal ratio. Recently, Zhang et al. identified AS-enriched species such as *Bacteroides* and *Parabacteroides* through metagenomic profiling [54]. Consistent with these findings, our sequencing results demonstrated that the predominant gut microbiota in AS primarily consisted of *Bacteroides* and *Parabacteroides* compared to other groups, and treatment with SCFAs was able to modulate the abundance of these microbes to resemble that of the control group. This suggests that SCFA supplementation may help restore a healthier gut microbiota composition in individuals with AS, potentially influencing disease progression and the complications associated with gut dysbiosis in this population.

In summary, our study has shed light on the potential role of gut dysbiosis in contributing to inflammation and secondary bone loss in ankylosing spondylitis (AS). The dysregulation of gut microbiota leading to gut barrier dysfunction appears to be a key factor triggering the activation of inflammation in AS. Our findings suggest that supplementation with short-chain fatty acids (SCFAs) may effectively reduce the expression of inflammatory cytokines, ameliorate the pathological changes in the intestine, joints, and spine, and modulate the composition of gut microbiota.

### Limitations and Benefits

Although our research results demonstrate the potential effects of short-chain fatty acids in alleviating the pathological changes associated with AS and modulating the gut microbiota, there are some limitations to our study. Firstly, our study was primarily based on animal models, thus requiring further clinical research to validate the applicability and effectiveness of these findings in humans. Secondly, while we observed the inhibitory effects of SCFAs on inflammatory factors, the extra control group needs further investigation to determine the exact therapeutic dose of BA. The experimental design was built upon previous research utilizing indole-3-acetic acid (IAA), a microbial tryptophan metabolite, to regulate the immune pathways and mitigate the inflammatory response in AS. In the previous experiment, the groups were divided into a control group, AS group, and IAA treatment group [22]. An additional vehicle control group is often considered essential for comparisons and analysis among different animal groups. We realized that the absence of a vehicle control group may limit the conclusions of this study. However, it is worth noting that many researchers investigating the therapeutic effects of butyric acid in various diseases have also adopted a three-group design: a control group, a disease model group, and a model group with butyrate supplementation. For instance, Huang et al. demonstrated that butyrate alleviated diabetic retinopathy by modulating the gut microbiota and enhancing the expression of tight junction proteins ZO-1 and Occludin [55]. Similarly, Zhou et al. examined the therapeutic effects of sodium butyrate in high-fat diet-induced steatohepatitis [56], and Zhao et al. showed that sodium butyrate reduced excessive inflammatory responses in rat models of chronic obstructive pulmonary disease [57]. Moving forward, our future project will include complete groups with a vehicle control group and different dose treatment groups. Additionally, a fold change basis may be the better method to prove all the above experiment indexes, but we will also ensure that all the experimental results, including the staining results and ELISA data, are presented relative to a control group, and further research is needed to delve into the detailed mechanisms of how SCFAs influence bone metabolism, immune responses, gut barrier function, and other aspects.

For a better understanding of the relationship between the gut microbiota and AS, future research can further explore the specific changes in the microbiota and their connection to the pathogenesis of AS, aiming to identify potential biomarkers for early diagnosis and personalized treatment of AS. Moreover, researchers can conduct more clinical trials to validate the therapeutic effects of SCFAs in AS patients and assess their long-term safety and sustainability. Ultimately, we hope that by deepening the understanding of the interaction between AS and the gut microbiota, scientific evidence can be provided for the development of more effective treatment strategies and interventions to improve the quality of life and prognosis of AS patients.

## Figures and Tables

**Table 1:** Primer sequence information of tight junction proteins in three groups.

Gene	Forward Primer (5′-3′)	Reverse Primer (5′-3′)
ZO-1	GATGAGCGGGCTACCTTA	TGGAGACTGCGTGGAATG
Occludin	CCTGGAGGTACTGGTCT	ATCTTTCTTCGGGTTTT

## Data Availability

The data presented in this study are available on request from the corresponding author.
